# Fluorescent silica nanoparticles as an internal marker in fruit flies and their effects on survivorship and fertility

**DOI:** 10.1038/s41598-022-24301-7

**Published:** 2022-11-17

**Authors:** Nut Songvorawit, Photchara Phengphuang, Thitinat Khongkhieo

**Affiliations:** Behavioural Ecology Laboratory, Department of Biology, Faculty of Science, Chulalongkorn University, Bangkok, 10330 Thailand

**Keywords:** Biological techniques, Nanoscience and technology

## Abstract

Tracking and differentiating small insects at the individual levels requires appropriate marking materials because of their small size. This study proposes and investigates the use of fluorescent silica nanoparticles (FSNPs) as an internal marker owing to their good optical properties and biocompatibility. FSNPs were prepared using the water-in-oil reverse microemulsion technique with Rubpy dye as a fluorophore. The obtained particles were spherical, monodispersed in nanosize and exhibited bright orange luminescence under ultraviolet (UV) light. Internal marking was accomplished in fruit flies (*Drosophila melanogaster*) through feeding. The result shows that the fruit flies exhibit bright luminescence in their abdomen when exposed to UV light. The marking persistence duration of FSNPs in the fruit fly bodies is longer than those of other fluorescent dyes. Fruit flies fed with FSNPs have a longer lifespan than those fed with Rubpy dye. There was no difference in fertility and negative geotaxis response among the treatment and control groups. These findings demonstrate that FSNPs can be used as an internal marker in fruit flies, and are possibly applied with other small insects with a translucent abdomen.

## Introduction

Marking is a crucial step in understanding the behavior, activities, and dispersal of actively mobile animals since it allows researchers to distinguish and identify them at the individual level^1^. Various techniques have been used for marking, depending on budget and target organisms^2^. Painting, tagging with bands or collars, mutilation, and tattooing are inexpensive and easy to implement^3–8^. Using more advanced technology, such as radio and satellite telemetry, offers more accurate data on animal movement and habitat use^9,10^. Some of these techniques have been applied to insects, but only to large species such as beetles, butterflies, bees, and dragonflies^11–16^. However, marking small insects is considerably more challenging owing to their size^9^.

Fluorescent marking is a technique used for small insects, where materials with luminescent properties are employed to mark outside or inside the targets. Individuals marked can be identified visually by their luminosity against a dark background when exposed to ultraviolet (UV) or short-wavelength light. Fluorescent dust is a conventional technique for marking small insects using particles that can glow under UV light. The procedure is straightforward: place the insects and dust in a container and shake until the dust sticks to the insect surface^17,18^. However, long-term studies might not be suitable because of the limited persistence lifetime, and too much dust may obstruct spiracles, affecting insect survival and behavior^1^.

Internal marking using fluorescent dye is another technique to overcome the drawbacks of fluorescent dust, where insects easily obtain the dye through feeding. This technique suits insects whose integument is thin and translucent enough to allow excitation and emission light to pass through^19,20^. Since dye molecules directly contact animal tissues and are possibly absorbed through the digestive tract, impacts on physiology are hardly inevitable and should be concerned^21^. Fluorescence quenching is another issue in which the fluorescence emission gradually fades out during the excitation period, mainly because of the reaction to oxygen in the surrounding environment^22,23^. This phenomenon limits the efficiency of fluorescent dyes when long-term observation is required.

Nanomaterials are currently gaining attention in biological science owing to their small size and unique properties. Quantum dots (QDs) and fluorescent silica nanoparticles (FSNPs) are two prominent types of fluorescent nanoparticles (NPs) that are useful for marking and labeling. QDs are semiconductor nanocrystals that emit bright luminescence under UV light^24^. Many colors are available depending on their elemental composition and size. External marking using QDs has been successfully used in *Trilobium castaneum*^25^. Additionally, using indirect marking, bee pollination has been tracked by labeling pollen with QDs^26,27^. However, most QDs are composed of heavy metals, especially cadmium, which is not environmentally friendly, and their toxicity to biological systems is still debated^24,28^.

FSNPs are another type of NPs in which numerous fluorophores are incorporated inside an amorphous matrix of silica particles, resulting in a significantly magnified optical signal compared to a single dye molecule^29^. Moreover, because the dye is contained within the silica matrix, which acts as an efficient barrier between the dye and the surrounding environment, the dye has great photostability^22,23^. Because of their great photostability, these nanoparticles are well suited for applications requiring high intensity or sustained excitations, such as detecting bacteria, cancer cells, and biomolecules^30–34^. However, there have been no reports of FSNPs being used as insect markers.

It is possible that using FSNPs as a fluorescent marking material in insects might be an effective approach. Therefore, this study evaluates the possibility of using FSNPs as a marker in small insects. The FSNPs are prepared, and their physical properties are characterized. The efficacy of marking and its influence on biological aspects are investigated using fruit flies, *Drosophila melanogaster*, as a model organism.

## Results

### Characteristics of FSNPs

Approximately 70 mg of dried FSNPs were obtained from each time of synthesis. Amount of fluorescent dye found in the supernatant after sysnthesis was very low (< 1% of total dye in the reaction system) which indicated that almost all of the dye molecules were trapped inside the NPs.

**Figure 1:**
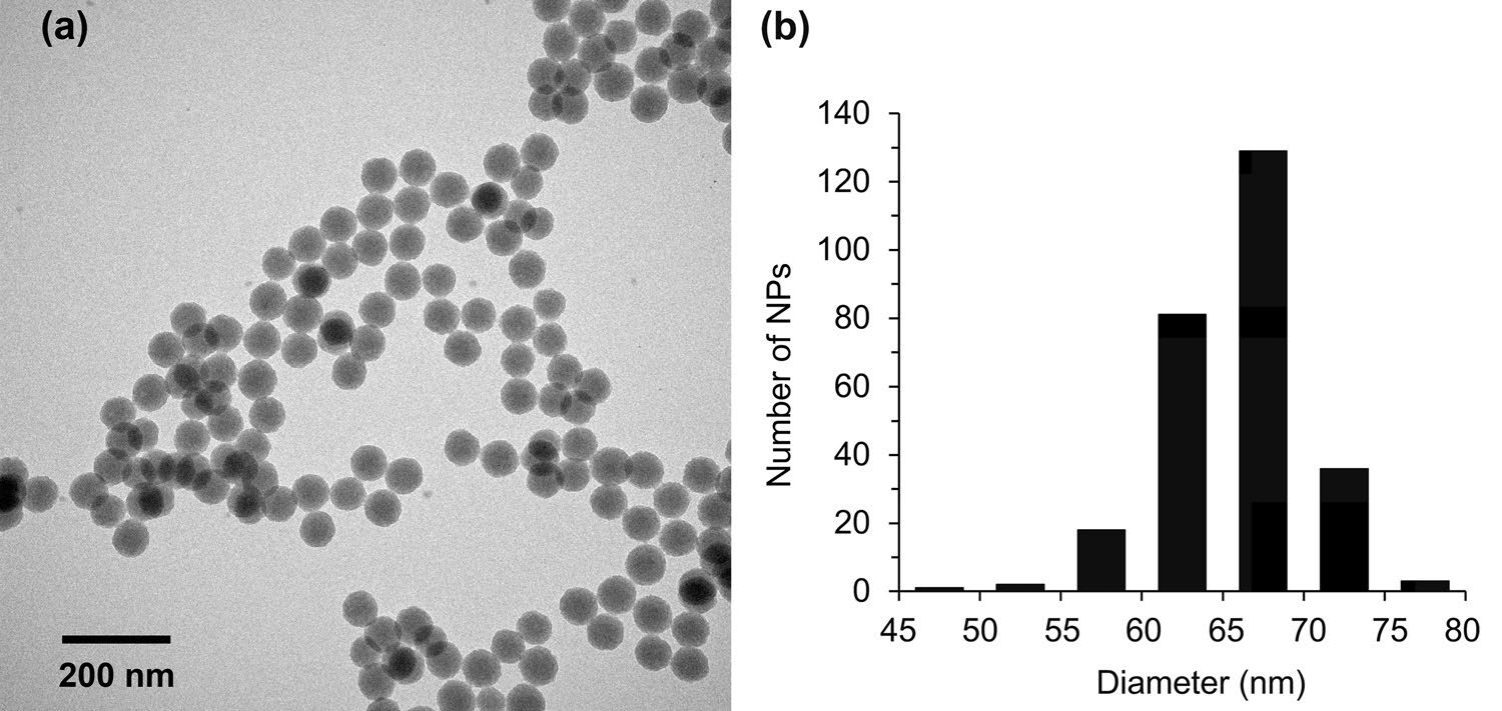
(**a**) A transmission electron microscope (TEM) image at 100,000 × magnification showing the spherical shape of FSNPs and (**b**) size distribution of FSNPs measured from TEM images using ImageJ software (version 1.53o, NIH, USA).

FSNPs were highly spherical and monodispersed (Fig. 1a), and NPs were uniform in size, with an average diameter of 65.78 ± 4.38 nm (Fig. 1b). True density of FSNPs was 1.763 g/cm^3^ and the concentration was 3.804 × 10^15^ particles/g of dried NPs. Based on calculation, the number of dye molecules trapped in a single NP was 43,407 molecules. The average zeta-potential of dispersed 1 mg/mL FSNPs in deionized water (pH = 5.8) was − 31.5 ± 0.76 mV (*n* = 6), indicating moderate stability. However, when FSNPs were dispersed in the liquid food for rearing fruit flies (pH = 5.8), zeta-potential increased to − 10.85 ± 0.40 mV (*n* = 6), indicating incipient instability.

**Figure 2:**
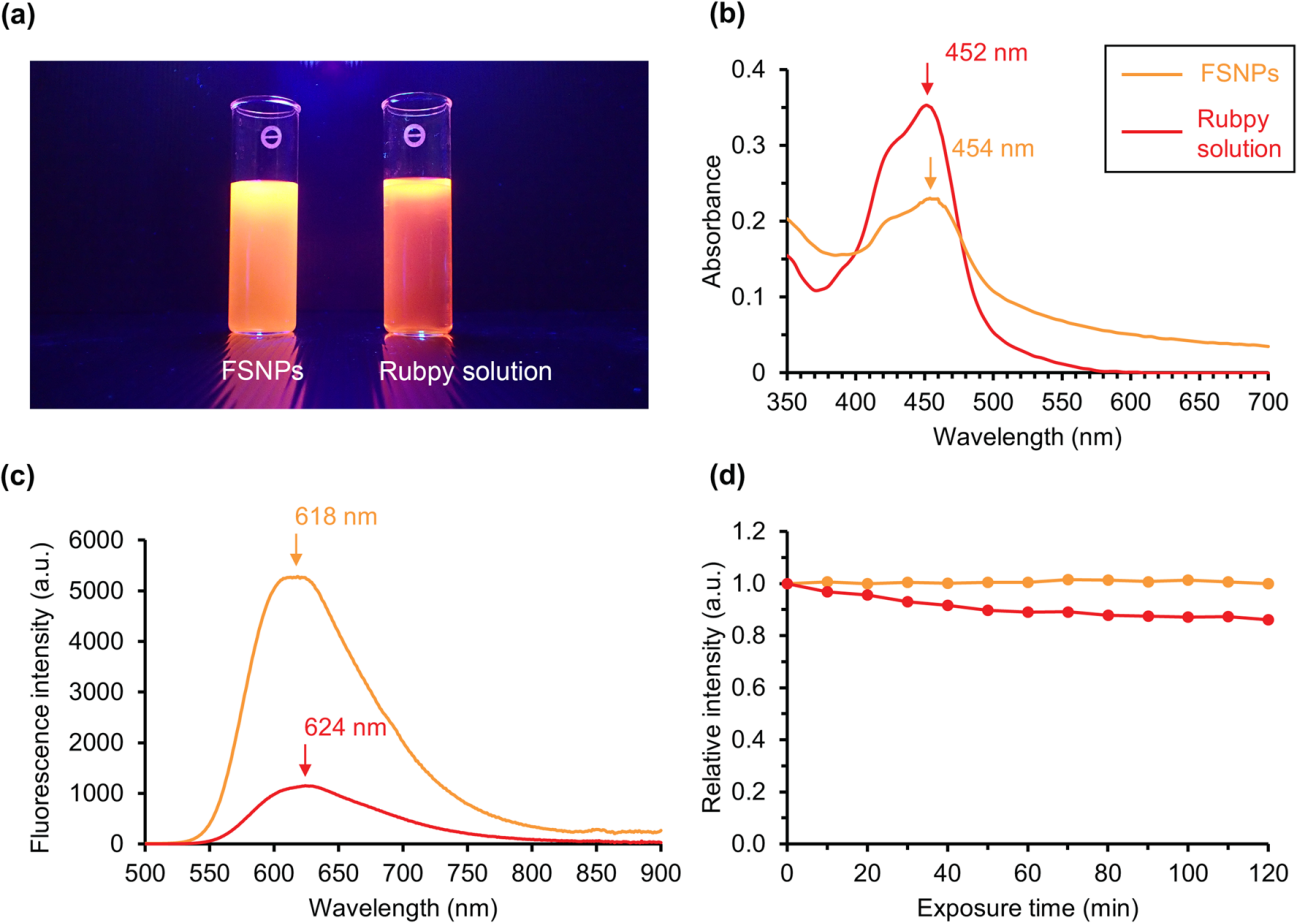
(**a**) Orange luminescence of FSNP suspension and Rubpy dye solution under UV light, (**b**) absorption spectra of 0.1 mg/mL FSNPs and 0.027 mM Rubpy dye solution, (**c**) emission spectra of 1 mg/mL FSNPs and 0.27 mM Rubpy dye solution when excited with 460 nm excitation band, and (**d**) fluorescence stability of 1 mg/mL FSNPs suspension compared to 0.27 mM Rubpy dye solution after being exposed to a 150 W mercury vapor lamp for 2 h. Arrows indicate maximal absorption or maximal emission wavelengths.

The FSNP suspensions glowed bright orange luminescence under UV light (Fig. 2a). Absorption spectra of FSNPs and the Rubpy dye solution was similar with maximal absorption wavelengths at 454 and 452 nm, respectively (Fig. 2b). Maximal emission wavelength of FSNPs was 618 nm which was slightly blue-shifted relative to that of the Rubpy solution (624 nm) under a 460 nm excitation band (Fig. 2c). However, the fluorescence intensity of FSNPs was higher at equal dye molar concentrations. FSNPs showed greater photostability than that of the Rubpy dye solution, with their fluorescence brightness changing very little over the course of 2 h of excitation under a 150 W mercury vapour lamp (Fig. 2d).

### Appropriate quantity of FSNPs for marking

**Figure 3:**
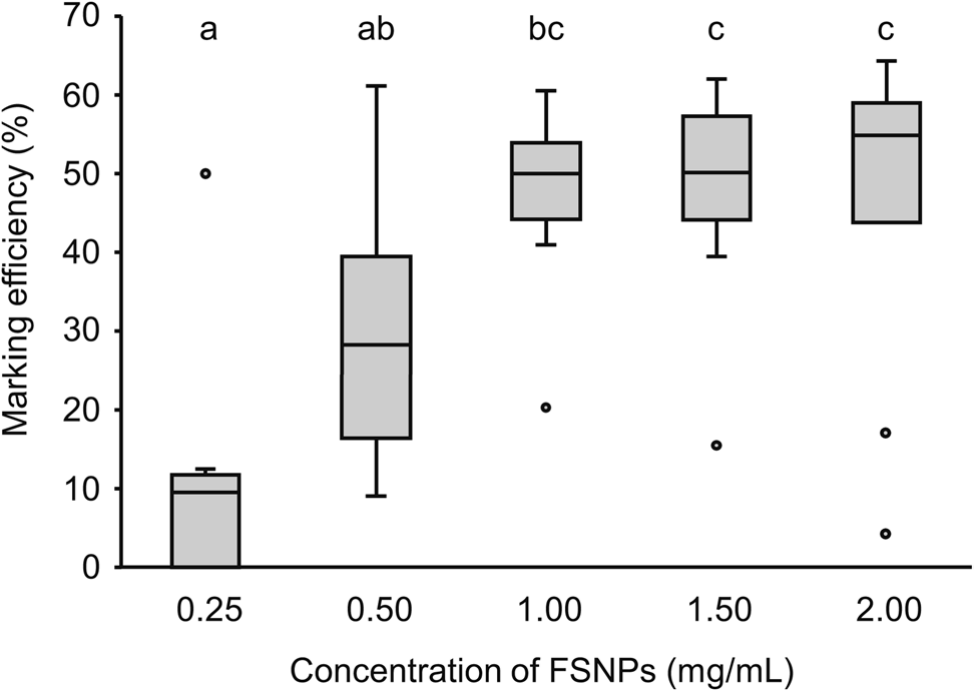
Marking the efficiency of FSNPs at different concentrations in *Drosophila melanogaster* (*n* of each treatment = 10, males and females combined). The horizontal lines within each box indicate the median, the box edges indicate the 75th and 25th percentiles, the whiskers show the maximum and minimum, and the circles represent outliers. Treatments with a common letter are not significantly different from each other as revealed by the aligned rank test at the 5% level of significance.

The marking efficiency varied significantly across NPs concentrations, increasing efficiency with increasing FSNP concentrations until 1.00 mg/mL (Fig. 3). The efficiency was not different between sex, and interactions between concentration and sex were not detected statistically (Table 1).

**Table 1:** Effects of markers, sex, and their interaction on marking parameters and characteristics of *Drosophila melanogaster*, using the aligned rank test.

Characteristics	Factors	df^a^	*F*^b^	*P*
Marking efficiency	FSNP concentration	4	9.89	< 0.001
	Sex	1	0.01	0.922
	FSNP concentration × sex	4	0.85	0.500
Feeding acceptance	Type of markers	3	1.35	0.259
	Sex	1	53.18	< 0.001
	Type of marker × sex	3	1.25	0.294
Marking persistence	Type of marker	2	27.39	< 0.001
	Sex	1	11.73	< 0.001
	Type of marker × sex	2	8.00	< 0.001
Longevity	Type of marker	3	95.37	< 0.001
	Sex	1	8.19	0.004
	Type of marker × sex	3	2.56	0.054
Climbing	Type of marker	3	1.19	0.317
	Sex	1	7.19	0.008
	Type of marker × sex	3	0.55	0.651

^a^df is the degrees of freedom.

^b^*F* is the F-statistic, which is the ratio of the mean sum of squares to the mean square error.

### Feeding acceptance

Feeding acceptance measured from the amount of food consumed indicated a significant difference between male and female flies, but not among marker types (Table 1). Average consumption of all tested foods ranged between 0.89 and 0.91 µL/individuals/day for males and between 0.94 and 1.03 µL/individuals/day for females.

### Marking persistence

**Figure 4:**
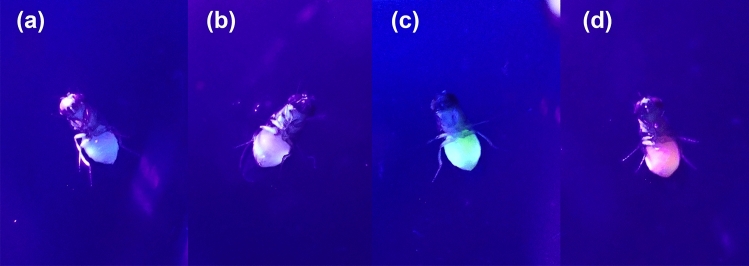
*Drosophila melanogaster* under a 40 W UV light bulb (365–400 nm wavelength) at a distance of 30 cm away after feeding liquid food containing different fluorescent markers for 1 h, (**a**) liquid food without a marker, (**b**) liquid food containing 0.27 mM Rubpy dye, (**c**) liquid food containing 0.27 mM fluorescein, and (**d**) liquid food containing 1 mg/mL FSNPs.

**Figure 5:**
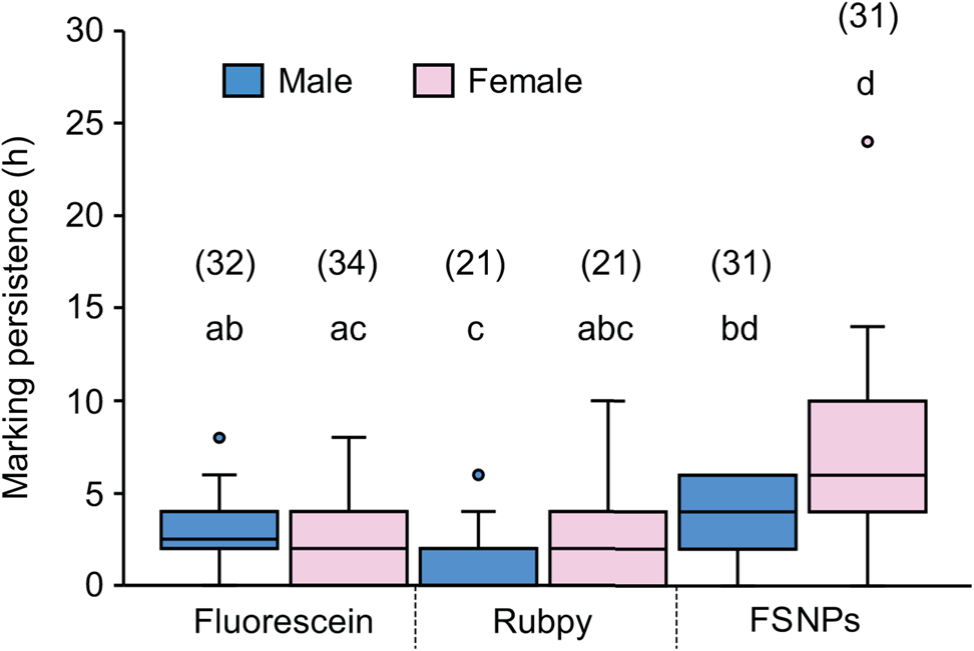
Detectable luminescence duration of *Drosophila melanogaster* after feeding liquid food containing different markers. The horizontal lines within each box indicate the median, the box edges indicate the 75th and 25th percentiles, the whiskers show the maximum and minimum, and the circles represent outliers. Treatments with a common letter are not significantly different from each other as revealed by the aligned rank test at the 5% level of significance. The numbers above the boxes are the sample size of each treatment.

Fluorescein- and FSNP-fed flies showed distinctively brighter luminescence in the abdomen than Rubpy-fed flies under UV light (Fig. 4). However, Rubpy-fed flies showed pale luminescence, which was difficult to distinguish from autofluorescence in non-marked flies. The duration of detectable luminescence from flies varied among marker types and sexes (Table 1). Luminescence from FSNP-fed flies could be detected longer than those with fed fluorescein and Rubpy dye (Fig. 5). However, luminescence in most flies did not persist longer than 10 h after feeding, except for some FSNP-fed females. During the experiment, bright fecal spots were noticed on the inner wall of vials from the first hour of feeding.

### Longevity

**Figure 6:**
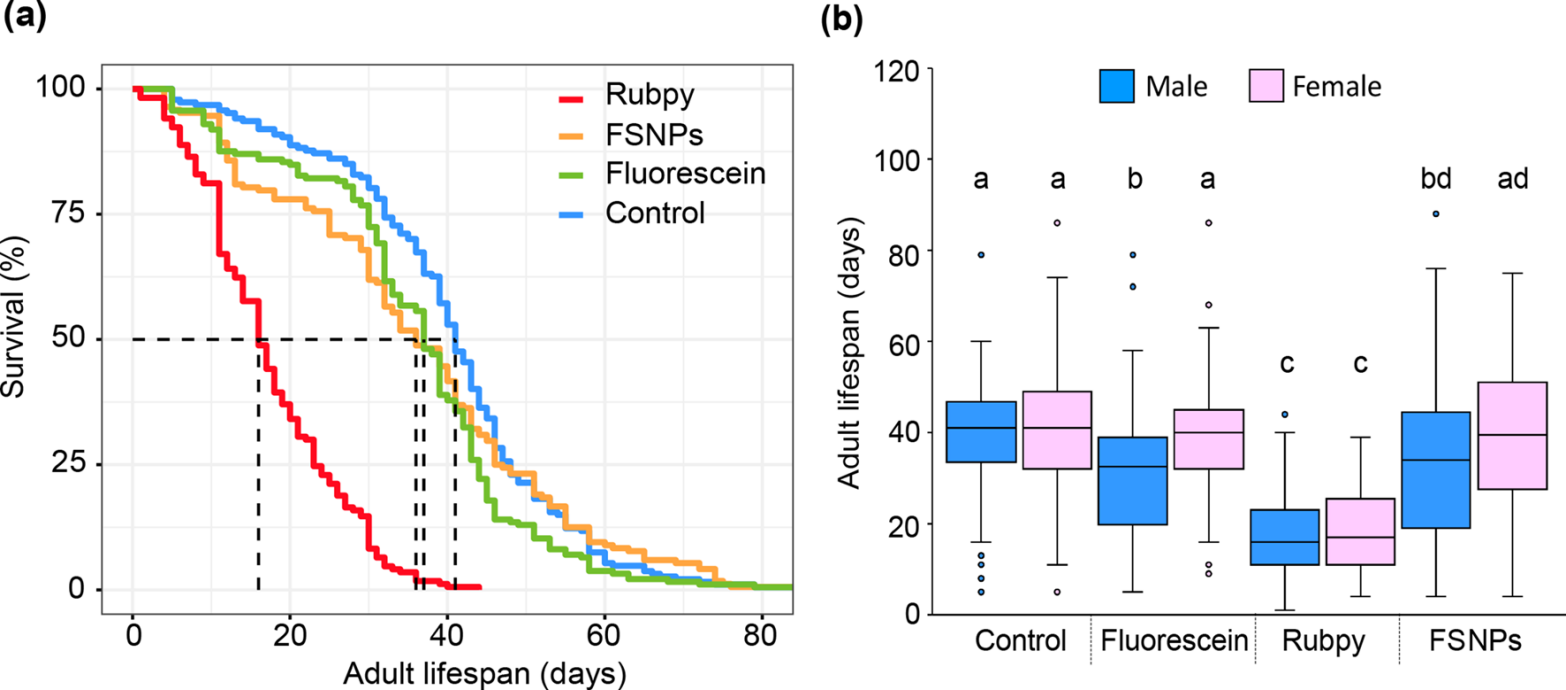
(**a**) Survival curves of *Drosophila melanogaster* fed with liquid food containing different markers and (**b**) lifespans of the fruit fly in each treatment. The horizontal lines within each box indicate the median, the box edges indicate the 75th and 25th percentiles, the whiskers show the maximum and minimum, and the circles represent outliers. Treatments with a common letter are not significantly different from each other as revealed by the aligned rank test at the 5% level of significance.

The effects of FSNPs on the longevity of adult flies were tested and compared with those of other dyes. Survivorship of fruit flies was significantly different among treatment groups (χ^2^ = 362, df = 3, *P* < 0.001). The survival probability of Rubpy-fed flies was lower than that of other groups of the same age (Fig. 6a). Overall, females had a slightly longer lifespan than males in all treatments. Median survival times of control, fluorescein-, Rubpy- and FSNP-fed flies were 41, 32.5, 16, and 34 days for males and 41, 40, 17, and 39.5 days for females, respectively (Fig. 6b).

### Fertility

The number of offspring developed into adults was highly varied but not significantly different among treated males, treated females, and untreated fly pairs (χ^2^ = 11.85, df = 6, *P* = 0.07) (Table 2). The offspring sex ratio of these tested pairs was slightly female-biased, and it was not significantly different among treatment groups (χ^2^ = 2.19, df = 6, *P* = 0.90) (Table 2).

**Table 2:** Fertility of *Drosophila melanogaster* males and females during 10 days-period after feeding different markers.

Treatment	Median number of adult offspring	Median offspring sex ratio
Normal male × normal female	107	0.45
Fluorescein-fed male × normal female	75	0.43
Rubpy-fed male × normal female	51.5	0.43
FSNP-fed male × normal female	56	0.46
Normal male × fluorescein-fed female	147	0.45
Normal male × Rubpy-fed female	122	0.44
Normal male × FSNP-fed female	76	0.44

### Negative geotaxis

The percentages of flies with negative geotaxis and the ability to traverse a 7 cm line in the control, fluorescein, Rubpy, and FSNP treatments were all 30% for males and 40%, 30%, 40%, and 50% for females, respectively. However, there was a significant difference between sex, but not among treatments (Table 1).

## Discussion

Bright luminescence, high photostability, and low toxicity are important features of fluorescent markers when applied to living organisms or biological systems^1,29^. In this study, the observed optical properties of FSNPs and free dye were slightly different. The shifts of absorption and emission peaks of FSNPs relative to those of free dye might be due to polarity of the medium surrounding dye molecules which is different between inner and outer sides of NPs^35^. The fluorescence signal from FSNPs was brighter than that of the Rubpy dye solution at the equivalent-molar concentration (Figs. 2c and 4). Excellent brightness of FSNPs has been reported previously^23,30,31^. Numerous fluorophores are incorporated inside an amorphous matrix of silica particles, resulting in a significantly magnified optical signal compared to a single dye molecule^29^. Silica nanostructures serve as a barrier protecting dye molecules from interacting with fluorescence quenchers (e.g. oxygen) in the surrounding environment, and thus maintaining the high fluorescence quantum yields^22,23,36^.

FSNPs did not appear to accumulate in the gut passage of fruit flies, and they were gradually excreted in the first hour after being consumed. The experiment in previous studies using FD&C Blue No. 1 mixed in the food indicated that fruit flies could excrete the food and dye consumed within 30 min^37,38^. This indicated that food retention time in the guts of fruit flies was very fast. However, females had longer marking persistence time than males (Table 1 and Fig. 5). This could be explained by the variation of gut sizes between sex in which females typically have larger guts than males, resulting in longer food and dyes (or NPs) retention time in the guts^39^. Nevertheless, the absorption of NPs can occur because of electrostatic interaction between the surface charge of NPs and the membrane in the guts. For example, under acidic conditions in the middle midgut of fruit flies, positively charged NPs can pass quickly while absorbed and stay longer in the alkaline posterior segment of the midgut^40,41^. The observation of these interactions has been achieved using fluorescent polymeric NPs^41^. According to zetapotential analysis in this study, surface of FSNPs was negatively charged which might have electrostatic between the NPs and the negatively charged cell membrane in the guts, and might also be influenced by van der Waals forces and hydrogen bonds^42^.

Not only are dye molecules protected from the surrounding environment, but silica nanostructure also prevents the direct contact between the dye and living cells, which could negatively affect the target organisms^29^. This could explain the rapid decline of fly survival in the Rubpy-fed group, whereas the flies treated with FSNPs lived longer and had a lifespan comparable to that of the control group (Fig. 6a). Although no studies on the toxicity of Rubpy dye in living organisms have been conducted, it is hypothesized that this fluorescent dye has a negative effect on fly survival and that silica nanostructure could prevent this effect.

There are still discrepancies regarding the toxicity of silica NPs. For example, amorphous silica NPs are currently being developed as food additives and drug-delivery agents owing to their safety for human health, low toxicity, and compatibility with biological systems^23,43–46^. However, it has been reported that FSNPs increased apoptosis of human lung epithelial cells and showed cytotoxicity in MRC-5 cells^47,48^. Even when only FSNPs were prepared using the same process and fluorescent dye (Rubpy dye) as in this study, reports of toxicity varied among tested organisms. For example, they had no negative effect on zebrafish embryos but promoted apoptosis in human lung epithelial cells^45,47^. Silica NPs seem to have distinctively entomocidal effects and are developed to be pesticides. Furthermore, when used as dust, they absorb water from insect cuticles and cause abrasion on the wax layer, eventually leading to death from desiccation^48–50^. *D. melanogaster* larvae exposed to silica NPs undergo adverse effects from gut membrane destabilization, mouthparts deformation, and oxidative stress^51,52^. However, note that the toxicity of silica NPs depends on many factors, including the preparation technique, functional groups on the particle surface, particle size, dosage, and target cells or organisms^53,54^.

The FSNPs in this study aggregate faster in the liquid food than in deionized water. This phenomenon was confirmed by zeta-potential, which showed that when the particles were dispersed in liquid food containing sugar and peptone, the value was close to zero. Aggregation is a concerning issue when NPs are applied in the aqueous phase. This problem can be prevented by modifying functional groups on particle surfaces, making them suitable for applications in biological systems^55^. Therefore, investigating the effects of surface modification in vivo is recommended for future studies.

## Conclusion

This study shows the achievement of using FSNPs synthesized using the water-in-oil reverse microemulsion (WORM) technique as an internal fluorescent marker in fruit flies. FSNPs were highly spherical and monodispersed, with an average diameter of 65.78 ± 4.38 nm. Fruit flies can easily intake the NPs through liquid food, yielding bright orange luminescence in the abdomen under UV light. Additionally, no adverse effects on survival and fertility were detected compared to the control group. The particle surface modification can be used to increase the persistence of NPs in the insect body or to design NPs with specific binding to target tissues/organs. More studies on the interactions between NPs and living organisms are required for future research.

## Methods

### Synthesis of FSNPs

FSNPs were prepared using the WORM technique with slight modifications from the previous studies by using Rubpy dye (tris (2,2′-bipyridyl)ruthenium(II) chloride) as a fluorophore due to well-embedded in the silica network, and there is no need of pre-modification of the dye molecules before NP synthesis^31,32,34,56^. Next, 15 mL cyclohexane, 3.6 mL 1-hexanol, 3.54 mL Triton™ X-100, and 0.96 mL of 20 mM Rubpy dye solution were mixed and stirred for 20 min. Then, 0.2 mL tetraethyl orthosilicate (TEOS) was added and stirred for 10 min. After the formation of WORM in the system, 0.2 mL of 28–30% ammonium solution was added to catalyze the reaction and stirred further for 24 h at room temperature under dark conditions.

To reduce dye leakage from NPs, the product was subsequently coated with a silica shell by repeating the reaction for one more cycle^57^. Then, 0.96 mL deionized water, 0.2 mL TEOS, and 0.2 mL of 28–30% ammonium solution were added to the slurry and stirred for 24 h. After the reaction was completed, FSNPs were separated from the solution with acetone precipitation and then washed with 95% ethanol and distilled water to remove the solvent and surfactant. Dye leakage was measured from the absorbance of the supernatant after precipitating the NP product at 460 nm using a spectrophotometer (Dlab, SP-V1000), and compared with the standard curve of Rubpy dye solution. The washed FSNPs were stored in a suspension in the dark at 4 °C.

### Characterization of FSNPs

FSNPs were examined under a transmission electron microscope (TEM; JEOL JEM-1400). The diameter was measured from TEM images (three independent samples, 90 particles per sample) using ImageJ software (version 1.53o, NIH, USA; https://imagej.nih.gov/ij). True density of NPs was measured by helium pycnometry using a Ultrapycnomter 1000 (Quantachrome Instruments). The aggregation of NPs in an aqueous solution was measured in terms of zeta-potential using a zeta-sizer (Malvern Panalytical, Nano ZSP). The higher the value (positive or negative), the more stable the particle dispersion, with 0 to ± 10 mV indicating that the NPs are unstable and tend to aggregate, ± 10 to ± 30 mV incipiently instable, ± 30 to ± 40 mV moderately stable, ± 40 to ± 60 mV well stable, and values beyond ± 60 mV excellently stable^58^. Absorption spectra were analyzed using a spectrophotometer (Dlab, SP-V1000), and emission spectra were analyzed using a spectrofluorometer (Shimadzu, RF-6000) at a maximal excitation wavelength of 460 nm^32^. To test fluorescence stability, 1 mg/mL of FSNP suspension was exposed to a 150 W mercury vapour lamp, and its fluorescence intensity was measured every 10 min for 2 h.

### FSNP concentration and the number of dye molecules embedded in a particle

Concentration of FSNPs was expressed as the number of NPs in 1 g of dried NPs, which was calculated from the formula for the volume of a sphere, as shown in Eq. (1):

1 $${V}_{NP}=\frac{4\pi {r}^{3}}{3}$$

where *V*_*NP*_ is the volume of a NP and *r* is the average radius of FSNPs in the unit of cm.

The number of FSNPs (*N*_*NP*_) in 1 g was calculated according to Eq. (2):

2 $${N}_{NP}=\frac{1}{(D \times {V}_{NP})}$$

where *D* is the true density of FSNPs in the unit of g/cm^3^.

To estimate the number of dye molecules trapped in a single NP, calculation was performed based on the assumption that all dye molecules were trapped inside FSNPs and each synthesis yielded 70 mg of dried NPs.

### Fly culture and husbandry

Wild-type fruit flies, *Drosophila melanogaster*, collected from natural populations in Bangkok, Thailand, were bred and maintained on a standard cornmeal-sucrose-yeast agar medium (100 g/L cornmeal, 50 g/L sucrose, 50 g/L yeast powder, 20 g/L agar, 0.65 g/L sodium propionate, and 0.1 g/L methylparaben) at 25 °C ± 1 °C, 60% ± 10% relative humidity under a 12/12 h light/dark cycle. This condition was used throughout the study.

### Appropriate FSNP concentration for marking

An appropriate concentration of FSNPs for marking fruit flies was measured in terms of marking efficiency. FSNPs were mixed in liquid food (100 g/L sucrose and 10 g/L yeast extract) and adjusted to five concentrations: 0.25, 0.5, 1.0, 1.5, and 2.0 mg/mL. Groups of 20–40 flies with 3 to 5 days of adult age were starved for 18–20 h in 240 mL glass bottles containing 1.5% water agar before the experiment. The experiment was conducted in 240 mL glass bottles with eight feeders made of 200 µL pipette tips hanging from the bottle lid (8 tips per bottle, loading 20 µL liquid food per tip). The flies were allowed to feed on the food for 2 h before being freeze-killed. The luminescence of flies due to FSNPs intake, which was visible through the cuticle of the abdomen, was visually inspected at a distance of 30 cm away from the flies under a 40 W UV light bulb (365–400 nm wavelength). Fluorescent brightness was scored ranging from 0 to 2: 0 = no fluorescence, 1 = slight fluorescence, and 2 = bright fluoscence. Marking efficiency was calculated following the formula described in a previous study^17^, as shown in Eq. (3):

3 $${\text{Marking efficiency (\% ) }} = { 100 } \times \frac{{{(}n_{F} \times {\text{b)}}}}{{{\text{(B}} \times N_{F} {)}}}.$$

where *n*_*F*_ is the number of flies with each score value, *b* is the brightness score, *B* is the highest possible score (here: 2), and *N*_*F*_ is the total number of scored flies. The experiment was conducted with five replications.

#### Feeding acceptance

Feeding acceptance was measured as the quantity of food intake through capillary feeders during a 3 day period^59^. Groups of five flies with 3–5 days of adult age were kept in 45 mL glass vials containing 1.5% water agar. Liquid food containing 1 mg/mL FSNPs was provided to flies through two 5 µL capillary feeders hanging from the cotton plug. After 24 h, the quantity of food intake was measured, and the old capillaries were replaced by new ones. The reduction of liquid food from evaporation was determined from feeders in the vials without flies. Feeding acceptance of FSNPs was compared with the feeding of other markers, i.e., liquid food containing 0.27 mM fluorescein, liquid food containing 0.27 mM Rubpy dye, and liquid food without marker (control). The concentration of dye solution at 0.27 mM was selected because this concentration was equivalent to the amount of dye presented in 1 mg/mL FSNPs based on calculation.

### Marking persistence

Fruit flies with 3–5 days of adult age were starved for 18–20 h in 45 mL glass vials containing 1.5% water agar. The test began at 08:00 h by allowing flies to feed on liquid food containing different markers as described earlier through feeders for 1 h. Then, the markers were replaced by normal liquid food. Luminescence from the flies was observed under UV light every hour until the luminescence was not detected. Flies without luminescence at the beginning (indicating no feeding) were excluded from the analysis.

### Longevity experiment

Groups of 10 flies consisting of five males and females with an adult age less than 12 h were kept in 45 mL glass vials containing 1.5% water agar. Liquid food containing different markers described earlier was provided to the flies ad libitum through 200 µL pipette tips and renewed every other day. The numbers of dead and alive flies were checked daily, and the live flies were transferred to new vials every week to prevent fungal infection until all individuals died.

### Effects on fertility

Newly emerged virgin flies (adult age less than 6 h) of the same sex were treated by rearing with tested food containing different markers in the same way as the longevity experiment for 7 days. To identify the effects of dyes or FSNPs on reproduction in relation to the sex, treated (fed with food containing the marker) and untreated (fed with normal food) flies were paired, i.e., treated male × untreated female and untreated male × treated female. The flies were reared with CYS agar one pair per vial and transferred to fresh vials every five days for 10 days. The vials were kept under standard rearing conditions until all offspring developed into adults. The number of offspring was counted, and the sex was identified.

### Negative geotaxis

The effects of markers on locomotion and behavioral response were investigated from negative geotaxis using a climbing assay. The rearing, food, and feeding apparatus were conducted similarly to the longevity experiment. After 2 weeks of the feeding tested foods, groups of 10 flies of the same sex were transferred to new glass vials, and the vials were marked at 7 cm above the bottom. Flies were left without disturbance for 1 h before conducting the behavioral test. Then, the flies were tapped to the bottom three times and given 10 s to climb upward to the top. The numbers of flies above and below the 7 cm mark were recorded. The experiment was repeated for three trials with 1 min intervals. Ten replications were conducted per treatment.

### Statistical analyses

The effects of markers, sex, and their interaction on marking parameters and characteristics of fruit flies were analyzed using an aligned rank test^60^. The effects of markers on longevity were visualized by plotting survival curves using Kaplan–Meier analysis, and differences in survival rates were tested using the log-rank test. The number of offspring and offspring sex ratio were compared among treatments using Kruskal–Wallis test. A *P* value < 0.05 was considered statistically significant. Statistical analyses were performed using the base, ARTool, survival, and survminer packages in R, version 4.1.3^61^.

## Data Availability

All data generated and analyzed within this study are available from the corresponding author upon reasonable request.
